# Effects of guanotrophication and warming on the abundance of green algae, cyanobacteria and microcystins in Lake Lesser Prespa, Greece

**DOI:** 10.1371/journal.pone.0229148

**Published:** 2020-03-11

**Authors:** Valentini Maliaka, Yvon J. M. Verstijnen, Elisabeth J. Faassen, Alfons J. P. Smolders, Miquel Lürling

**Affiliations:** 1 Aquatic Ecology and Water Quality Management Group, Department of Environmental Sciences, Wageningen University, Wageningen, The Netherlands; 2 Department of Aquatic Ecology and Environmental Biology, Institute for Water and Wetland Research, Radboud University, Nijmegen, The Netherlands; 3 Society for the Protection of Prespa, Agios Germanos, Greece; 4 B-WARE Research Centre, Radboud University, Nijmegen, The Netherlands; 5 Research Institute RIKILT, BU Contaminants & Toxins, Wageningen University, Wageningen, The Netherlands; 6 Department of Aquatic Ecology, Netherlands Institute of Ecology, Wageningen, The Netherlands; INRA, FRANCE

## Abstract

Lake Lesser Prespa in Greece is a vital breeding habitat for the Dalmatian and Great White Pelican and a shelter for numerous rare and endemic species. However, eutrophication processes are distressing the lake system and the outbreaks of cyanobacterial blooms during the warm months may pose a threat to aquatic organisms due to the presence of microcystins (MCs). In this study we hypothesize that nutrients (eutrophication), nutrient-rich pelican droppings (guanotrophication) and warming (climate change) can affect the algal growth and MCs production in the water layer of Lake Lesser Prespa. Seston collected from three lake sites was incubated at ambient (20°C) and high (30°C) temperature with or without the addition of nutrients (nitrogen (N), phosphorus (P)), or pelican droppings. Results showed increased chlorophyll-a at higher temperature (30°C). N addition yielded higher chlorophyll-a levels than the non-treated water or when only P was added. The addition of both N and P as well as the addition of pelican dropping resulted in the highest chlorophyll-a at both temperatures. Notably, in the dropping-treatments, cyanobacteria and MCs were promoted while changes were evoked in the relative contribution of toxic MC-variants. Guanotrophication may thus influence the cyanobacterial dynamics and most likely their toxicity profile at Lesser Prespa.

## Introduction

The shallow Lake Lesser Prespa (also known as Mikri Prespa) in NW Greece (40°41'N, 21°37'E) is part of the wider Prespa tri-national basin, which includes Lake Greater Prespa and is shared by Greece, Albania and the Former Yugoslav Republic of Macedonia (SW Balkans, SE Europe). This interconnected freshwater system has been nominated as the first transboundary protected area in the Balkans [1] and has further received numerous national and international designations [2]. Particularly, Lake Lesser Prespa (47.4 km^2^) is a Ramsar Wetland of International Importance and a Special Protected Area (Directive 79/409/EEC) due to the exceptional endemic biodiversity and the important populations of endangered and vulnerable species [3]. The breeding avifauna includes a remarkable colony of Great White Pelicans (*Pelecanus onocrotalus*) and the largest colony of Dalmatian Pelicans (*Pelecanus crispus)* in Greece and worldwide (20% of global population) [2,4–6]. The populations of both species within the Lesser Prespa area show a linear increase over the last decades (circa 500–600% increase between 1991 and 2016) [5,7]. Likewise, the number of breeding pairs of the Great and Pygmy cormorants (*Phalacrocorax carbo*, *Microcarbo pygmeus*), in Lesser Prespa have, respectively, doubled and tripled since 1991 [5].

Apart from its high ecological value, this iconic lake and its surroundings offer other key ecosystem services such as fisheries, recreation and water provisioning for irrigation [3]. Since the 1970s, bean farming is the most prevalent agricultural activity within the Lesser Prespa area [8]. Early limnological assessments emphasized that the frequent supply of fertilizers and the use of unsustainable irrigation practices (furrow irrigation) have caused a progressive eutrophication of the lake [8–11]. In 1993 the irrigative area of the Lesser Prespa area was nearly all used for bean cultivation (ca. 1100 ha), while the population in the area was circa >1000 to 2000 inhabitants [12]. The mesotrophic—eutrophic status of the lake is reflected in elevated chlorophyll-a levels during warm periods [13,14], as well as in the relatively high level of nutrients in the lake water column and the decreased dissolved oxygen concentrations in the bottom waters [9,11]. Cyanophytes (*Microcystis* sp.) are dominant within the phytoplankton community and the outbreaks of cyanobacterial blooms have been sporadically recorded in the lake during in summer and early autumn in recent years when hepatotoxic microcystin (MC) was detected in potentially harmful levels [15]. Besides bean farming or other anthropogenic nutrient inputs, waterbirds may also act as an important vector of nutrients. In the Lesser Prespa area concerns are expressed about the potential effects of waterbirds on the water quality since they occur in high abundances and may stimulate algal production via their excrements which is rich in N and P [16–18]. This may especially occur nearby their breeding and roosting sites (guanotrophication) [16,19].

Rising temperatures and eutrophication can mutually reinforce the excessive growth of algae in freshwater lakes and stimulate the growth of cyanobacterial species [20,21]. Numerous experimental findings show that warmer conditions and nutrient enrichment may favor potentially toxic strains of cyanobacteria and affect not only the MC concentration levels but also their synthesis [22–25]. In consequence, future warmer climate and excessive nutrient run-offs associated with extreme weather events (i.e heavy rainfalls) are expected to enhance the growth of nuisance cyanobacteria in freshwaters systems which can pose a severe risk to environmental and human health due to their potent toxins [26,27]. However, MC concentrations in surface waters do not necessarily correlate with the number of potentially toxic cyanobacterial cells, because many environmental variables are influencing the dynamics of toxic and non -toxic cyanobacteria, as well as the intracellular MC concentrations [28–30].

This paper presents and discusses the experimental findings from an algal bioassay where the effects of nutrient-rich pelican droppings (as a proxy of guanotrophication) and warming on the algal growth potential were studied by using seston from Lake Lesser Prespa. Comparably, the effect of eutrophication on algal growth was also tested by increasing the availability of solely nitrogen (+N), solely phosphorus (+P) and both nitrogen and phosphorus (N+P). An earlier algal bioassay in autumn 2013 showed that algal growth in Lake Lesser Prespa and a nearby small pond (called Vromolimni) is N-limited as well as N and P co-limited [15]. Likewise, MC concentrations increased at higher N and both N and P availability while the most toxic variant MC-LR only was promoted at higher temperatures.

In this study we hypothesize that algae growth will be promoted in water enriched with nutrients as well as in water enriched with nutrient-rich pelican droppings. Additionally, the effect of warming (20°C, 30°C) was tested for seston incubated with the different above mentioned nutrient treatments and expected to enhance the growth of algae and cyanobacteria. Moreover, increased MC levels may accompany the potential intensification of cyanobacterial growth under the aforementioned conditions.

## Materials and methods

**Fig 1:**
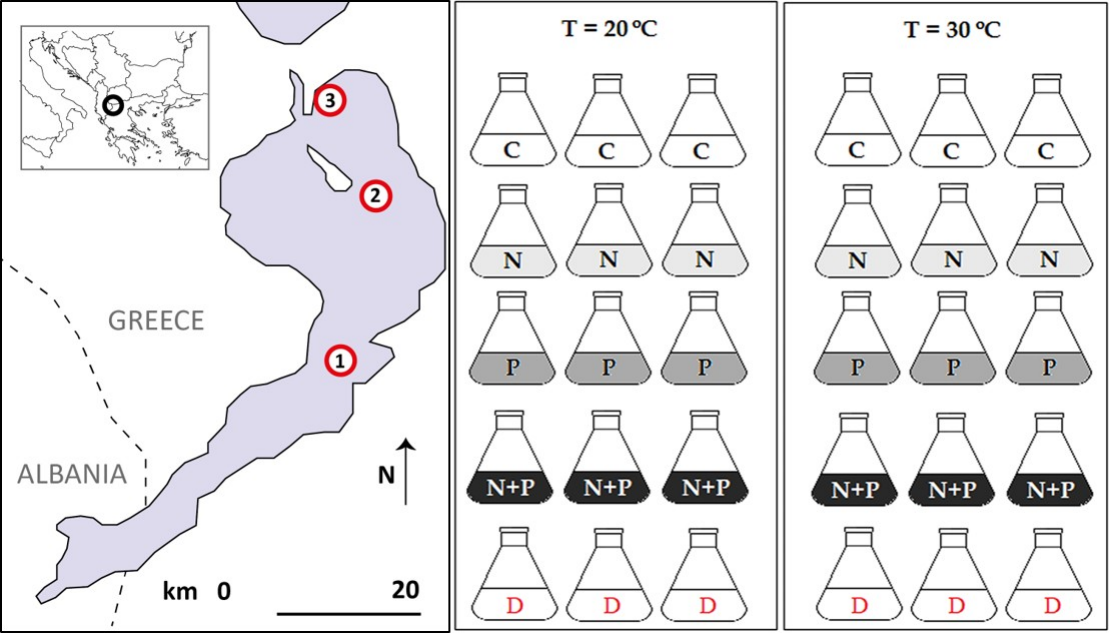
Experimental set-up. (A) Locations of water sampling sites at Lake Lesser Prespa in 2015 (site 1, 2, 3). (B) the set-up of the algal bioassay per sampling site; per site 30 flasks were incubated at two different temperatures (20 and 30°C) and received five different nutrient treatments—no nutrient addition/control (C), nitrogen addition (+N), phosphorus addition (+P), addition of both nitrogen and phosphorus (+N+P) and addition of pelican droppings (D), all in triplicate.

Lake Lesser Prespa is an ancient lake located in the Prespa basin, south of Lake Great Prespa. Lesser Prespa has a surface area of circa 47.4 km^2^, a maximal depth of 8.4 m (average depth 4.1 m) and lies at a mean altitude of about 850 meter above sea level [31,32]. The total catchment of the basin is around 189 km^2^ and the residence time of the water has been reported to be 4.8 years [31]. In 1990–1991 the annual phosphate concentration was 0.2 μmol L^-1^ and nitrate+ammonium concentration was 11.4 μmol L^-1^. In October 2015 similar nutrient concentrations were found (this study). In the Lesser Prespa area two pelican colonies breed namely: the Dalmatian Pelican with an abundance of >1100 breeding pairs in 2008–2012 and 500 pairs of Great White Pelicans in 2011–2012 [5], aside with cormorants and herons. The pelicans breed during spring/summer at the northern part of the lake [2]. The algal growth bioassay was performed by using natural surface water (seston) collected from three sites at the Greek part of Lake Lesser Prespa on October 30^th^ 2015 (sites 1, 2 and 3; Fig 1). Sampling permit was required for the water sampling. The permit for water sampling at Lake Lesser Prespa was obtained from the Prespa National Park Management Body (Prespa, Greece).

### Field sampling

The depth at the sites 1 (40°44'7.48"N, 21° 5'24.79"E), 2 (40°46'38.96"N, 21° 6'23.54"E) and 3 (40°47'59.82"N, 21° 4'58.33"E) was circa 7 m, 5.5 and 1.8 m respectively. At the shallow site 3, a rather dense cover of submerged macrophytes was observed (*Myriophyllum spicatum*, *Ceratophyllum demersum*), while at the two deeper sites 1 and 2 no macrophytes were present. At each site two liters of surface water (top 0.5 m) were collected with a Rüttner sampler and stored in HDPE bottles. Per site, a glass-fiber filtered subsample (Whatman GF/C, Whatman international, Ltd., UK) was treated with 0.1 ml of HgCl_2_ (1 g L^-1^) after filtration in order to inhibit microbial alteration prior to analysis. Filtered samples were stored in the freezer (-20°C) and analyzed colorimetrically for dissolved phosphorus and dissolved inorganic nitrogen (ammonium and nitrate) using an Auto Analyzer 3 system (Bran+Luebbe, Germany) at Radboud University Nijmegen.

### Algal bioassay

The water samples were stored in a cool box and transported within 48 hours to the Netherlands. The algal bioassay was set up at the Aquatic Ecology & Water Quality Management Group (Wageningen University) three days after sample collection. For each sampling site (Sites 1, 2 and 3), five nutrient treatments were performed in triplicate: 30 Erlenmeyer flasks of 100 ml were filled with 50 ml surface water (seston) (Fig 1). Concerning nutrient treatments: 6 flasks received no nutrients (Control), 6 flasks received phosphorus (1.54 mg P L^-1^ as K_2_HPO_4_, +P treatment), 6 flasks received nitrogen (14 mg N L^-1^ as NaNO_3,_ +N treatment), 6 flasks received both phosphorus and nitrogen (1.54 mg P L^-1^ as K_2_HPO_4_ and 14 mg N L^-1^ as NaNO_3,_ +N+P treatment) (concentrations represent conditions that should alleviate any limitation of the respective element [33]) and the other 6 flasks received 5.1 mg dry weight (DW) of pelican droppings each, which resulted in a concentration of 0.10 g dried droppings L^-1^ (Dropping treatment). Prior to the Dropping-treatment, the nutrient content of the pelican droppings collected at Prespa area was analysed (see **‘***Pelican dropping analysis’*). The amount of pelican droppings added (based on water soluble phosphate contents of the droppings) to each Dropping-treated flask (circa 5.1 mg DW) corresponded to the amount of P added to the P-treated flasks. All flasks were incubated (SANYO Electric Co., Ltd., Osaka, Japan) with light supply of 120 μmol m^2^–^1^ sec^-1^ in 18:6 light:dark cycles and continuously shaken at 60 rpm, for seven days. A short term experiment was chosen to investigate the initial algal growth response. One week incubations are long enough to evoke clear responses in phytoplankton to nutrient enrichment using seston from a waterbody [34]. Biotic effects were expected to possibly only play a minor role in algal biomass on this short term. Visual inspection of the seston did not reveal presence of large-bodied cladoceran grazers, but ciliates, rotifers will have been present as well as different algae. For each treatment half of the flaks were incubated at 20°C and the others at 30°C. At the start of the bioassay (t = 0) and after seven days of incubation cyanobacterial and eukaryote algae chlorophyll (green algae, diatoms) were measured using a PHYTO-PAM (PHYTO-ED, system II version, Heinz Walz GmbH, Effeltrich, Germany), which has a strong capacity to distinguish between these major phytoplankton groups [31]. In a recent study of Lürling et al. [34] PHYTO-PAM measurements were tested thoroughly and in general the results were quite fair. Only when for instance *Planktothrix rubescens* is part of the cyanobacterial community, the PHYTO-PAM will partly place it in the blue channel and partly in the brown channel [34]. A selection (n = 22) of aliquots of 25 ml from Control flasks (C), treated flasks with combined P and N (+N+P) and flasks treated with pelican droppings (Droppings) were filtered over a glass-microfiber filter (Whatman GF/C, Buckinghamshire, UK). Due to limited availability of the MC analysis equipment this selection was made for MC analysis targeting on dropping treatments that resulted in cyanobacterial growth complimented with +N+P and control flasks with no/little cyanobacteria present. Filters with seston were stored in 8 ml glass tubes at -20°C and used for microcystin (MC) analysis (see *Microcystin and nodularin analysis using LC-MS/MS*).

An additional algal bioassay was done in order to verify if the pelican droppings contained any residue of cyanobacteria or other algae groups, which could therefore contribute to algal growth. Six Erlenmeyer flasks were filled with 50 ml of WC medium (enrichment suitable for algae growth [33]); 3 flasks served as control (no bird droppings added) and to 3 flasks circa 5.1 mg dry weight of pelican dropping was added. All flasks were incubated at 20°C with the same settings as the abovementioned algal bioassay. The same process was repeated with 6 flasks incubated at 30°C. Cyanobacterial and eukaryote algae chlorophyll were measured using a PHYTO-PAM at the start of the bioassay (t = 0) and after seven days of incubation.

### Microcystin and nodularin analysis using LC-MS/MS

The frozen filters with seston taken from the algal bioassay at the end of the incubation period (day = 7) were extracted and processed for microcystin (MC) analysis at the Netherlands Institute of Ecology as described by Lürling and Faassen [35]. All samples were analyzed for eight MC variants (dm-7-MC-RR, MC-RR, MC-YR, dm-7-MC-LR, MC-LR, MC-LY, MC-LW and MC-LF) and nodularin (NOD) by Liquid Chromatography with tandem Mass Spectrometry detection (LC-MS/MS) as described in [35]. Information on limit of detection and limit of quantification concentrations in samples analyzed for Microcystins is shown in S1 Table in the Appendix and other performance characteristics of the method are given in [35].

### Pelican dropping analysis

Fresh pelican droppings (n = 10) were collected at a platform which lies nearshore at Lake Great Prespa (Greece) which is a roosting spot for Dalmatian Pelicans. Dissolved ortho-phosphate and dissolved inorganic nitrogen (NO_3_^-^, NH_4_^+^) contents and other nutrients in pelican droppings were determined using a water-extraction method. A homogenized portion of circa 1 g of fresh pelican dropping was mixed with 100 ml milliQ water and the supernatant was analyzed colorimetrically using an AA3 system (Bran+Luebbe, Germany). For other elements inductively coupled plasma spectrophotometry was used (ICP-OES; Radboud University Nijmegen, The Netherlands). See for details of the analyses: van Dijk et al. [36]. Average concentrations of dissolved inorganic nitrogen were 9.65 ± 1.1 mg N g DW^-1^ and of ortho-phosphate 15.10 ± 2.6 mg P g DW^-1^ (S2 Table).

### Data analysis

End cyanobacterial and eukaryote algae chlorophyll concentrations of the algal bioassay were tested using SigmaPlot 12.0. A Two-Way ANOVA was conducted per site including the factors ‘nutrient treatment’ and ‘temperature’ (post-hoc Holm-Sidak). The data of site 2 were normally distributed, whereas data of site 1 and site 3 were log-transformed to establish normal distribution prior to further analysis. For the analysis of the microcystins, One-Way ANOVA was conducted for testing possible significant differences between the nutrient-treatments. A Two-Way ANOVA was used for the dropping treatment’s microcystin concentrations including the factors ‘site’ and ‘temperature’ (post-hoc Holm-Sidak).

## Results

### Initial chlorophyll-a and nutrient concentrations

**Fig 2:**
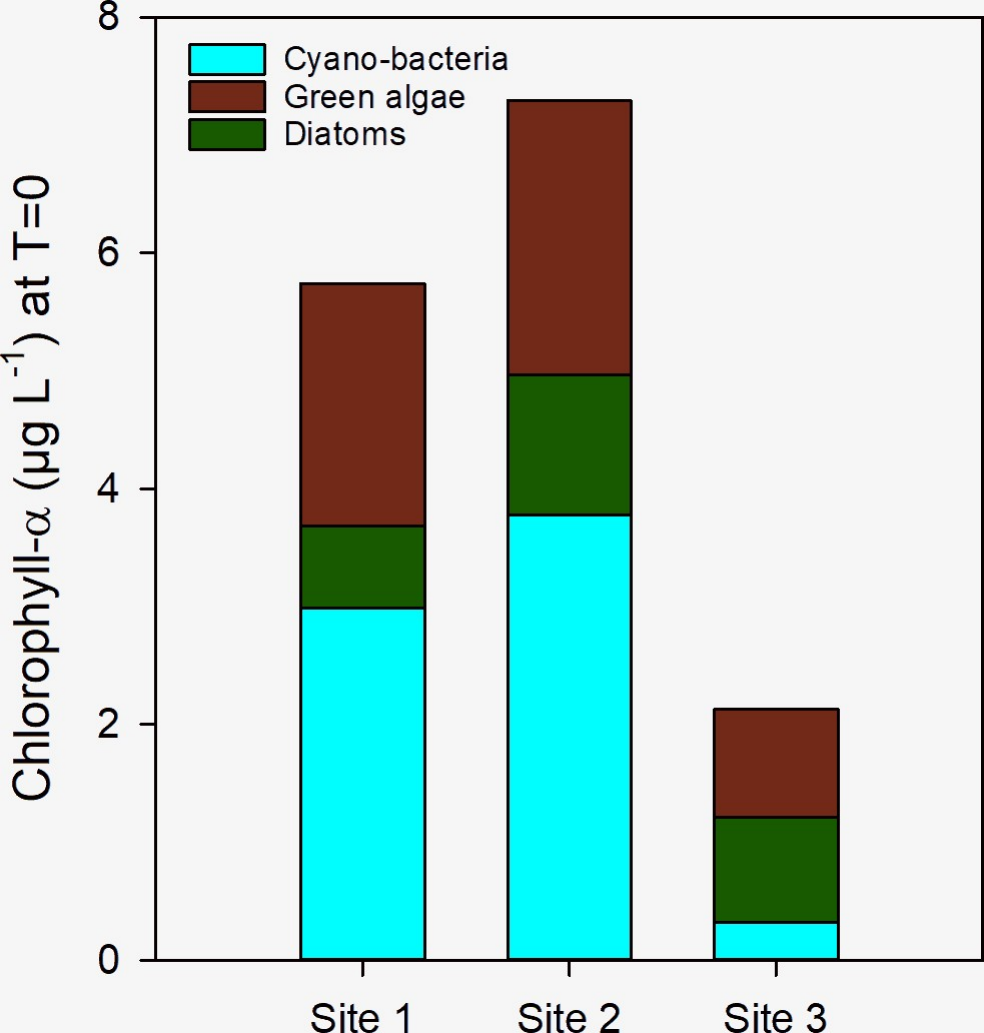
Initial chlorophyll-a concentrations (μg L^-1^) at t = 0 of Lake Lesser Prespa. Blue = cyanobacteria, Green = green algae, Brown = Diatoms, as measured at sites 1–3.

At the start of the incubation period (t = 0), the initial mean chlorophyll-a concentration measured with the PHYTO-PAM in samples from Lake Lesser Prespa varied between 2.1 μg L^-1^ (Site 3) and 7.3 μg L^-1^ (Site 2) (Fig 2). Cyanobacterial chlorophyll-a contributed for 52% to the total chlorophyll-a concentration in water from site 1 and site 2, but only for 15% in water from site 3 (Fig 2). The brown channel (diatoms) was the second abundant type of chlorophyll-a in sites 1 and 2 (31.8–35.7%), whereas green algae contributed 12.3 and 16.3%, respectively. At site 3, brown chlorophyll was highest (43.2%) followed by green algae (41.8%) (Fig 2). The initial ortho-phosphate concentrations were 0.2, 0.1 and 0.3 μmol L^-1^ and initial inorganic nitrogen (ammonium+nitrate) concentrations were 3.8, 7.8 and 3.3 μmol L^-1^ for sites 1, 2 and 3 respectively. Nutrient concentrations were measured again in each flask after the experiment (S3 Table). In all treatments ortho-phosphate and inorganic nitrogen were present. Lowest concentrations of phosphate were still present in control and +N treatment and for nitrogen lowest concentrations were found in control and +P treatments.

### Final chlorophyll-a concentrations

**Fig 3:**
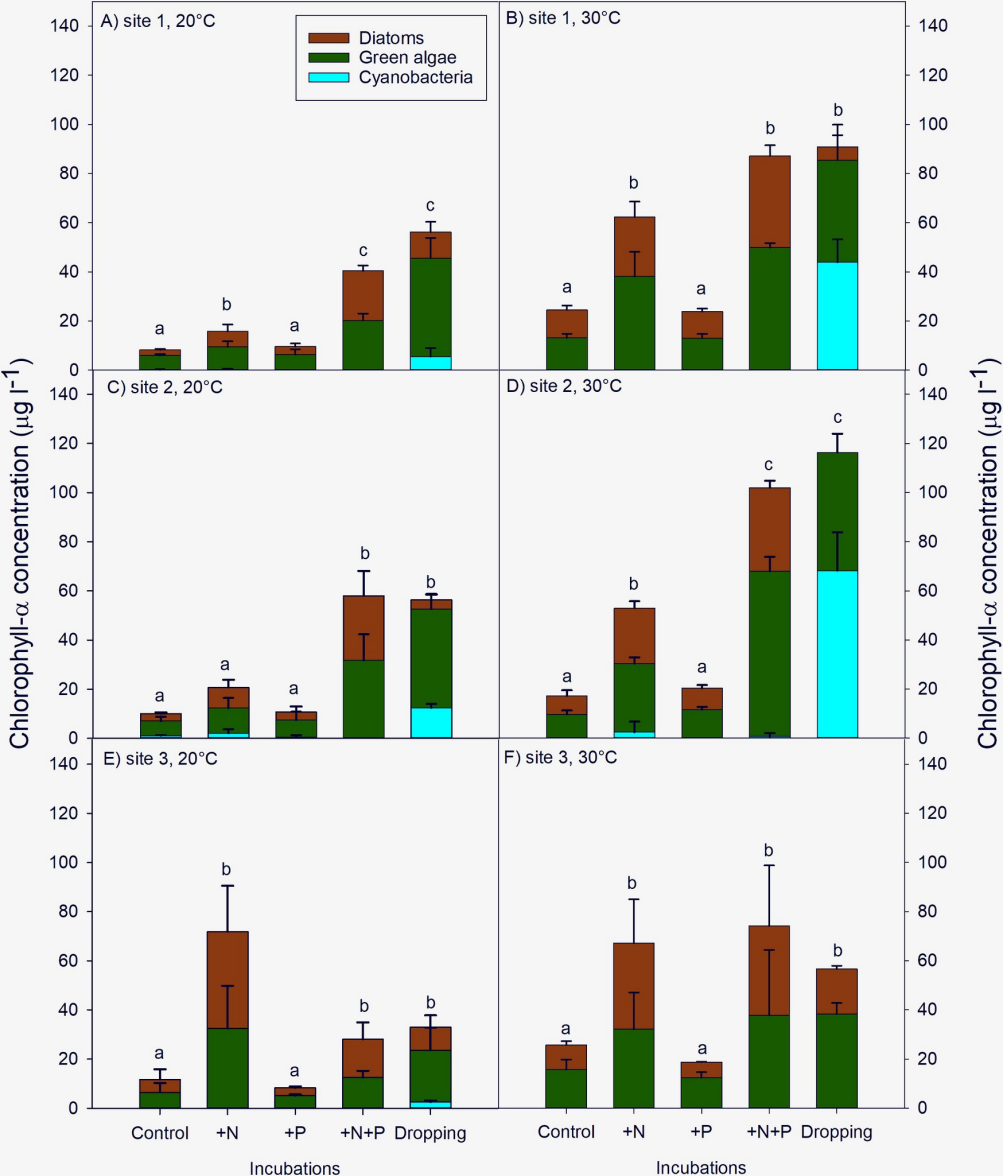
**Cyanobacterial (blue), green algae (green), and diatoms (brown) chlorophyll-a.** Concentrations as measured with PHYTO-PAM (μg L^-1^, mean ± standard deviation) at surface water sampled from three locations (Site 1,2,3) in Lake Lesser Prespa. The water had been incubated for seven days at 20°C and 30°C, without extra nutrients added (Control), with solely N-added (+N), solely P-added (+P), both N+P added (+N+P) and pelican droppings added (Dropping). Error bars indicate the standard deviation (n = 3). Similar letters above the bars indicate homogeneous groups per panel (total chlorophyll-a) that are not different at the 95% level.

After seven days of incubation at 20°C or 30°C, the chlorophyll-a concentrations in the +N and +N+P treatments were much higher compared to the control and +P treatment (Fig 3). In all cases chlorophyll levels in +P treated water were comparable to the controls. The water to which pelican dropping was added had end-chlorophyll-a levels which were comparable to the +N+P treated water. Although cyanobacterial chlorophyll was most abundant at start of the experiment (Fig 2), virtually no cyanobacteria chlorophyll-a was detected in controls and treatments, with the exception of the treatments with pelican droppings (Fig 3).

Addition of pelican dropping (Dropping treatment) led to a strong growth of cyanobacteria in water from the lake sites 1 and 2 and a decline or minor growth of diatoms, especially at a temperature of 30°C (Fig 3). Hardly any cyanobacterial chlorophyll was detected in the incubated water from site 3 (Fig 3). For all sites and nutrient treatments chlorophyll-a concentrations were significantly higher at 30°C compared to 20°C (p<0.05).

For site 1, a Two-Way ANOVA indicated significantly higher total-chlorophyll-a levels at 30°C than at 20°C (F_1,29_ = 151.3; p<0.001). A temperature x treatment interaction was present (F_4,29_ = 4.1; p = 0.014;). At both temperatures, +N+P and Dropping treatment were not significantly different, while control and +P treatments were not significantly different from each other either. The +N treatment had a significantly higher chlorophyll-a concentration than control and +P treatments, and a lower concentration than the +N+P and Dropping treatments at 20°C, but was similar to the +N+P and Dropping treatments at 30°C (Fig 3).

For site 2, there were significant effects of temperature (F_1,29_ = 59.1; p<0.001) and nutrient treatment (F_4,29_ = 61.1; p<0.001), while there was also a significant temperature x nutrient treatment interaction (F_4,29_ = 6.4; p = 0.002) for the total chlorophyll-a concentration. Chlorophyll-a concentrations were higher at 30°C than at 20°C. At 20°C, control, +N and +P treatments showed no significant differences (Fig 3). However, chlorophyll-a concentrations in these treatments were all significantly lower than the +N+P and Dropping (pelican dropping) treatment. The +N+P and Dropping treatments were not significantly different from each other. At 30°C, the +N treatment is significantly higher than the control and +P, but significantly lower than the +N+P and +dropping treatments.

For site 3, chlorophyll-a concentrations at 30°C were higher than at 20°C (F_1,29_ = 17.7; p<0.001) and significantly influenced by the treatments (F_4,29_ = 17.6; p<0.001). In contrast to the other sites, there is no interaction between temperature and treatment. At both temperatures, the same homogenous groups are observed, control and + P treatment form one group, while chlorophyll-a concentrations are significantly higher in the other group comprised of the +N, +N+P and Dropping treatments (Fig 3).

### Supplementary algal bioassay with WC-medium

In the additional experiment where WC-medium was used (see Materials and Methods) the initial chlorophyll-a levels (t = 0) were 0.06 μg L^-1^, 0.01 μg L^-1^ and 0.04 μg L^-1^ for cyanobacteria, green algae and diatoms, respectively. The total end-chlorophyll-a concentrations did not exceed 0.30 μg L^-1^ for both Control and Dropping treatments at both temperatures.

### Algal growth rates

**Fig 4:**
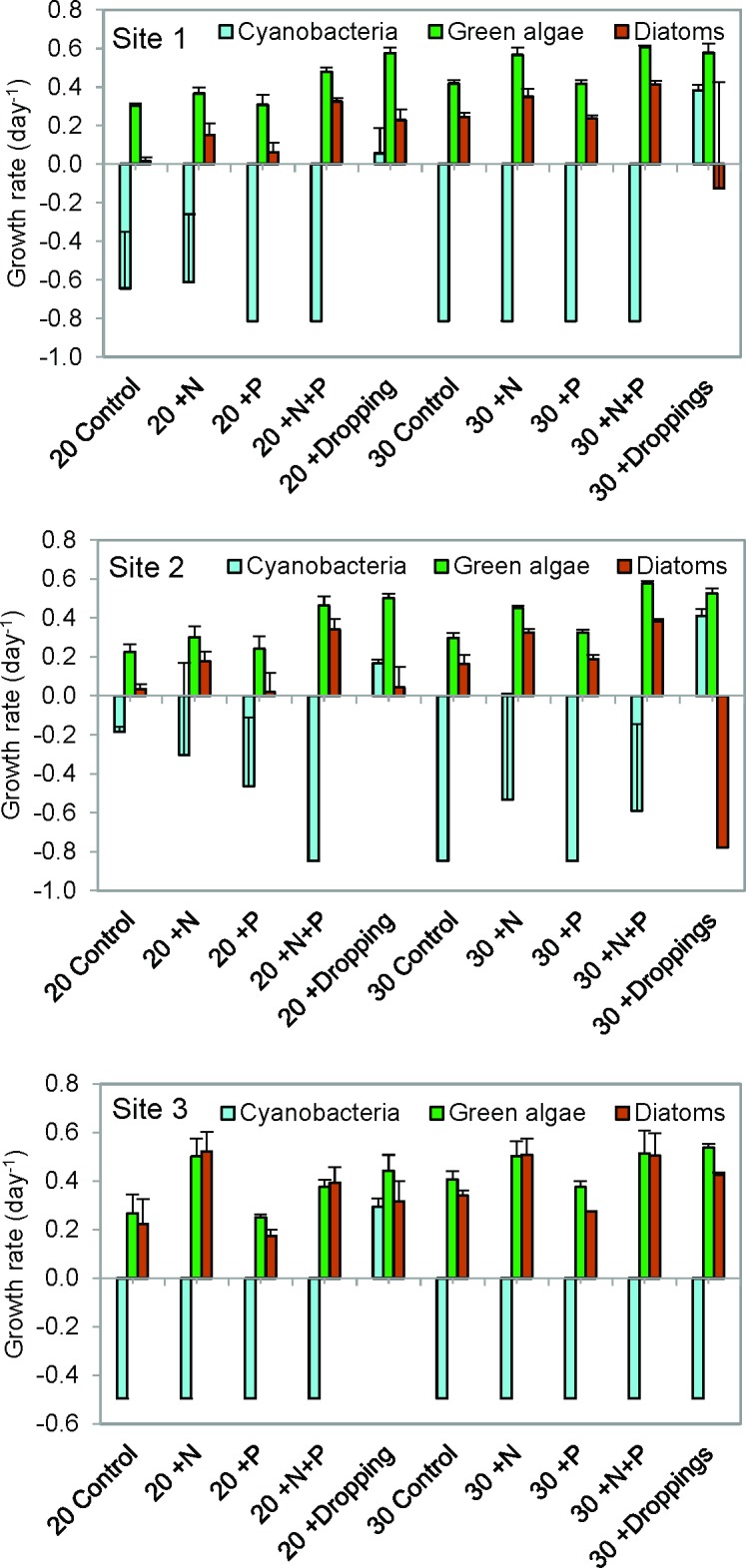
Algal growth rates in the algal bioassay. Growth rates (day^-1^) for cyanobacteria (blue), green algae (green) and diatoms (brown) based on PHYTO-PAM chlorophyll-a concentrations (μg L^-1^) in surface water sampled from three locations (Sites 1, 2 and 3) in Lake Lesser Prespa. The water had been incubated for seven days at 20°C and 30°C, without extra nutrients added (Control), with solely N-added (+N), solely P-added (+P), both N+P added (+N+P) and pelican droppings added (Dropping). Error bars indicate the standard deviation (n = 3).

After seven days of incubation, chlorophyll-a based growth rates (day^-1^, based on initial and end-chlorophyll levels and timespan of seven days) for cyanobacteria show that the dropping treatment stimulated the growth of cyanobacteria at all sites especially at warmer conditions (Fig 4). For all sites, green algae growth rates tend to be the highest in the treatments with +N, +N+P and Dropping. A similar trend is observed for the diatom growth rate for site 1 and 3. Overall, eukaryotic algal growth rates were positive (increase of chlorophyll) and higher than the cyanobacterial growth rates for all three sites (Fig 4).

### Final Microcystins (MC) concentrations

**Fig 5:**
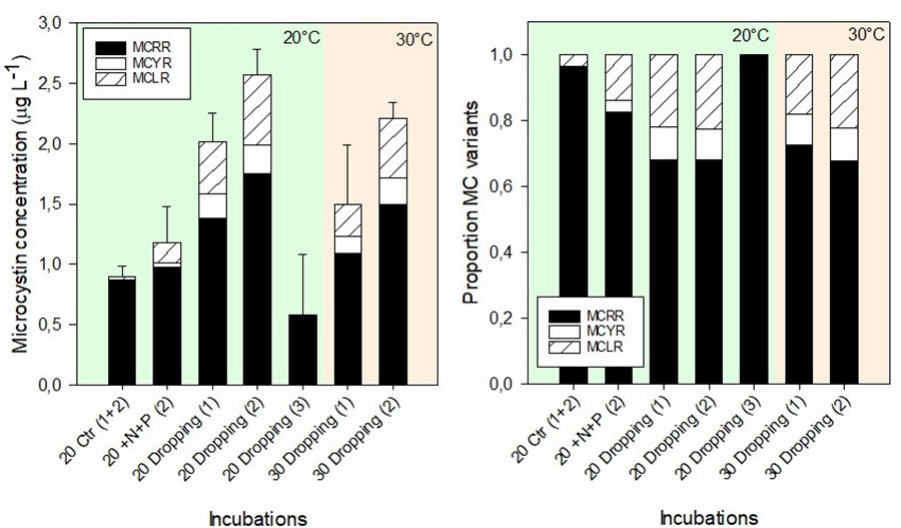
Microcystin concentrations in several treatments. (A) Concentrations of three microcystin (MC) variants (μg L^-1^) and (B) mean proportions of three different MC variants in the total MC pool in surface water sampled from three locations (Site 1,2,3) in Lake Lesser Prespa. The water had been incubated for seven days at 20°C and 30°C, without extra nutrients added (Control), with solely N-added (+N), solely P-added (+P), both N+P added (+N+P) and pelican droppings added (Dropping). Error bars indicate one standard deviation (n = 4 for Control (site 1 and 2), n = 3 for others).

Microcystins (MC) were present in the analyzed selected samples, reaching a maximum total concentration of 2.64 μg L^-1^ (Dropping treatment, site 2 at 20°C) (Fig 5A). Three MC variants were detected: MC-RR, MC-YR and MC-LR (Fig 5A and 5B). MCs were present in the control replicates (n = 4 at 20°C), although negligible amounts of cyanobacterial chlorophyll-a were detected in these samples (Fig 3). In all treatments, the most dominant MC variant was MC-RR comprising up to 100% (Dropping, site 3 at 20°C) of the total MC concentrations (Fig 5B). The dropping treatment at both temperatures for site 1 and 2 resulted in higher concentrations of MC-YR and MC-LR compared to the control or +N+P treatment (Fig 5A). In these dropping treatments the proportional contribution of MC-YR and MC-LR were higher than control and +N+P treatment as well (Fig 5B). By using One-way ANOVA, no significant differences were found (F_2,21_ = 3.150, p = 0.066) in the total microcystin concentrations between the treatments (Control 0.90 ± 0.08 μg L^-1^, +N+P 1.17 ± 0.30 μg L^-1^, Dropping 1.77 ± 0.77 μg L^-1^). However, there is a tendency towards significance. Two-way ANOVA for only the Dropping treatments (site 1 and 2) revealed a significant difference between the sites (F_1,11_ = 12.5; p = 0.008) and temperatures (F_1,11_ = 6.1; p = 0.036), but no interaction was present (F_1,11_ = 0.2; p = 0.683).

One-way ANOVA revealed significant differences in MC-LR (F_4,21_ = 15.587, p < 0.001) and MC-RR (p < 0.001) concentrations (temperatures were pooled) between treatments. There were no significant differences in concentrations between control, +N+P treatment and Dropping treatment site 3. Dropping treatment site 2 had for both MC’s the highest concentrations. For the MC-YR variant, One-way analysis on ranks showed that the concentrations in Dropping treatment site 2, were significantly higher than the control and Dropping site 3 (H_4_ = 17.235, p = 0.002).

The comparison of MC concentrations relative to total cyanobacterial chlorophyll-a between the treatments, reveals the highest ratio -higher than 1- for the control treatment (1.28 ± 0.08, Table 1), which means that MC-analysis yielded on average higher concentrations of MC’s than cyano-chlorophyll-a measured by PHYTOPAM in the control. For the +N+P treatment, no cyanobacterial chlorophyll-a was detected by PHYTO-PAM, though MC analysis of the analyzed +N+P samples did yield MC’s. The Dropping treatments show higher ratios at 20°C than at 30°C, where site 1 had the highest ratio (0.62) (Table 1).

**Table 1:** Total microcystin (MC) to cyanobacterial chlorophyll-a ratios (mean ± 1 SD in μg μg^-1^).

Treatment	MC:Chlorophyll-a
Control, 20°C	1.28 ± 0.75
+N+P, 20°C	-
Dropping (Site 2), 20°C	0.21 ± 0.03
Dropping (Site 1), 20°C	0.62 ± 0.62
Dropping (Site 3), 20°C	0.20 ± 0.17
Dropping (Site 2), 30°C	0.03 ± 0.01
Dropping (Site 1), 30°C	0.04 ± 0.02

## Discussion

### Effect of guanotrophication and warming on cyanobacterial growth

In the northern part of Lesser Prespa there is a high abundance of breeding piscivorous pelicans in summer [2,5] and as their excrements are rich in nutrients they can locally contribute to algal growth [16,17,19]. The experimental enrichment of surface water, collected from three different sites of Lake Lesser Prespa, with nutrient-rich pelican dropping indeed stimulated the algal growth and especially the profusion of cyanobacteria (Figs 3 and 4). Similarly, the simultaneous addition of nitrogen and phosphorus (+N+P treatment) produced strong algal growth (Fig 3). Apart from the co-limitation of N and P, at the sample locations the primary productivity is also nitrogen limited as the +N treatment led to significantly higher chlorophyll-a levels compared to the treatments in which no N (control and +P treatment) had been added (Fig 3). An earlier algal bioassay with surface water collected from Lake Lesser Prespa, September 2013 [15], and a field study conducted in 1999 [32] also showed algal growth to be limited by nitrogen. Although freshwater systems were often found to be P-limited [37] a more recent meta-analysis revealed that N and P limitations are equally important within freshwater systems [38]. The TIN/PO4 ratio of the initial seston already suggested N-limitation for sites 1 and 3 (ratio of ca. 4.6 weight based), according to the ratios found in [39]. Denitrification processes may remove large amounts of bioavailable N from aquatic systems, a process which is stimulated by higher temperatures [40,41] and may thus cause N limitation [31]. However, it is argued by Kosten et al. [42] that land use, catchment characteristics and hydrology are more critical for possible N-limitations in lakes than temperature. Furthermore, nutrient limitation may vary and shift over time and an algal community response to certain nutrient loads can be influenced by biotic factors such as zooplankton biomass or size [43].

It is possible that the N-limitation in Lake Lesser Prespa is caused by relatively high phosphorus input by agricultural run-off and domestic effluents [44]. As bean has a high phosphorus requirement [45], fertilizers used by bean farmers in Prespa area contain relatively high amounts of P to N, as discussed by Maliaka et al. [15]. In short, fertilizers used are YaraMila^™^ (12% N, 11% P_2_O_5_), TimacAgro Duofertil (11% N, 10% P_2_O_5_) and other Yara formulations (11% N, 15% P_2_O_5_) that have N:P ratios of about 2.5, which is evidently less than the Redfield N:P ratio of 7:1 [46]. Nutrients are transported to the lake via frequent surface irrigation of the bean fields and nutrient concentrations can be high in the drainage streams at the eastern part of the watershed. Next, water bird-derived nutrients can also represent a considerable input of nutrients to the lake, especially near breeding colonies [16–18]. Bird dropping can also be relatively more enriched in P than in N and therefore breeding colonies can, especially on a local scale, contribute importantly to the P-loading [17]. The pelican dropping analysis (**S3 Table**) in this study showed more directly available P than directly available N.

The incubation experiment of this study shows that the addition of dropping of the piscivorous Dalmatian Pelican (0.10 g dried dropping L^-1^) to the lake water may stimulate algal growth (Figs 2 and 4). Likewise, Bosman et al. [47] revealed that guano-enriched sea water led to higher algal growth compared to non-enriched sea water in a field experiment. Liu et al. [48] found that within the first days after droppings (of a goose) entered the water, circa 60% of the N—and 40% of the P content leached into the water. The addition of pelican droppings also leads to an increase of directly available nutrients. The additional experiment showed hardly any algae growth after adding fresh pelican dropping in WC-medium and therefore it can be assumed that the droppings do not contain viable algae residues. Nevertheless, the composition of algae was different in the pelican dropping treated replicates compared to other nutrient treatments. In most cases, cyanobacterial growth was stimulated in the water treated with pelican droppings (Fig 3), while other nutrient treated replicates did not show cyanobacterial prevalence. In the field, small amounts of fecal coliform bacteria have been associated with the proliferation of cyanobacterial blooms [49]. Possibly, the composition of the droppings promote conditions which are favorable for cyanobacteria, giving them an advantage when competing with green algae and diatoms. The availability of (micro-) nutrients, organic N and organic P, ammonium (which was the main form of inorganic N in the droppings), metabolites, vitamins or microbial matter in the droppings could have affected algal growth. Potassium (K), being an important nutrient next to N and P for algae (e.g. [50,51]), can also be available in high amounts in the pelican droppings (in our experiment 9.34 mg g DW^-1^). Also other elements were present within the droppings, which were not added in the nutrient treatments (e.g. sulphur, silicon, iron, **S3 Table**).

A temperature effect was expected as algal biomass often increases with higher temperature in light saturated systems [21,52]. Warming can affect the availability of nutrients in lakes [20,21,53], changing the dynamics [52]. In Lesser Prespa a mean annual temperature of 16.7°C, with a median maximum of 26.7 in July and August, was recorded in 2012–2013 [54], while an annual mean of 13.1 was found in 1990–1991 [13]. In the nutrient treatments, the added nutrients may compensate the increased resource demand [55], as can happen in warming lakes by e.g. extra mineralization in the catchment or sediment [21]. Though in the control treatment (no extra nutrients added), chlorophyll-a increased as well at 30°C, suggesting that the phytoplankton were still in their optimal growth range for temperature.

In meta-analyses, different outcomes of warming lake water on phytoplankton biomass have been found [53,55]. Kraemer et al. [55] found that (large) lakes with relatively low median chlorophyll-a tended to have more negative correlations between chlorophyll and lake water temperature, whereas lakes with relatively high median chlorophyll concentrations tend to show more positive correlations, possibly due to an increase in cyanobacteria. It seems that cyanobacterial chlorophyll was indeed promoted at the higher temperatures in the current study (sites 1 and 2), but only in the Dropping treatment. This is in line with previous findings that the share of cyanobacteria increases in warming freshwater systems because cyanobacteria have their growth optimum at higher temperatures and due to a decrease in grazing pressure as cyanobacteria are often less edible than green algae [20,26,55].

### Microcystins (MC) production and synthesis

The addition of pelican dropping material to the lake water seems to increase the MC concentrations to some extent compared to the control and to the replicates where both N and P were added (Fig 5). Remarkably, MCs were found in the control and +N+P treatment while only small amounts (control) or none (N+P treatment) of cyanobacterial chlorophyll was found in the PHYTO-PAM measurements. This suggests PHYTO-PAM measurements might have underestimated the cyanobacterial biomass in the lower ranges. The MC relative to cyanobacterial chlorophyll-a concentrations (MC:chlorophyll-a) differ strongly between the treatments but are well within the ranges published by Hollister and Kreaky [56].

In several bioassays, nitrogen additions (nitrate, ammonium or urea) stimulated chlorophyll-a and enhanced the MC concentrations [57]. The effect of water bird dropping on MCs, however, has rarely been studied in a comparable experimental set-up. MC analysis revealed three variants present in the incubated lake waters, namely MC-RR, MC-LR and MC-YR. MC-RR was the most abundant variant, while the relative contribution of MC-LR and MC-YR showed an increase in the dropping-treated waters (Fig 5A). Likewise, in a similar algal bioassay with water from Lake Lesser Prespa as well as the nearby pond Vromolimni, the addition of solely N or +N+P promoted the more toxic MC-LR at the expense of the RR variant [15]. Nutrient limitation can affect cyanobacterial growth and MC production. Nutrient analysis after seven days showed that P as well as N were non-limiting factors, except for the control treatment (S2 Table), which nevertheless showed a relatively high MC concentration.

Other studies have found a positive correlation between temperature and total MC concentration [22,58] or a change in toxin diversity with increasing temperature [59]. The results of the present study did not show an MC increase with higher temperature when treating the water pelican droppings. Although the MC levels were not significantly different, cyano-chlorophyll a levels were much higher at 30°C. As a result MC:chlorophyll-a declined in the dropping treatment at 30°C compared to the treatment at 20°C (Table 1). Lürling et al. [22] also saw a similar pattern when adding both N and P in a laboratory experiment to lake water. MC concentrations are a resultant of the cyanobacterial biomass of toxin producing cells and their MC cell quotas, where these cell quotas are not only determined by the presence of the synthesizing genes, but also by environmental factors that influence MC synthesis or MC fate [60]. The results suggest that the higher temperature stimulated the growth of non-MC producing or less toxic strains and/or lowered the MC cell quota of the toxic strains that were initially present. Furthermore, the composition of MCs in site 3 with dropping addition differs from the composition at the other two sites (1 and 2) (Fig 5A). This might be attributed to the possible changes in strain composition or heterogeneity in the initial cyanobacterial composition in the field [61,62]. Besides MC composition, the results of the algal growth test (end-biomass, -composition) of site 3 were different from site 1 and 2. Initial cyano-chlorophyll-a level was lower which resulted in lower end chlorophyll-a at the dropping treated flasks (Fig 3). Site 3 is located near the reed beds in a shallow area, where the macrophyte cover was also higher than at the deeper sites 1 and 2. These variables might have affected the initial conditions of the seston.

## Conclusions

The addition of pelican dropping material in surface water from Lake Lesser Prespa did significantly increase the algal biomass compared to the control or addition of solely N or P at 20°C as well as at 30°C. The addition of combined N and P (+N+P treatment) resulted in similar chlorophyll-a concentrations as in the Dropping treatments at the end of the incubation period. Overall, the relative contribution of cyanobacteria was higher in the dropping treatment at regular and high temperature compared to control and the nutrient treatments. Furthermore, the results of this experimental study showed that the algal growth in the lake was N-limited and N+P co-limited, as N addition yielded more biomass than P addition, and the simultaneous +N+P- addition led to a higher biomass than the treatment with solely N or P. Warming (20°C versus 30°C) did significantly increase algal biomass. Finally, MC concentrations increased after the addition of pelican droppings, while the (more toxic) variants MC-LR and MC-YR were promoted at the expense of MC-RR.

We conclude that in the case of Lake Lesser Prespa, guanotrophication can locally lead to serious algae blooms by increasing the availability of nutrients and that global warming may aggravate these blooms by promoting the growth of cyanobacteria. At the same time however, warming may lead to a lower production of toxic microcystins variants.

## Supporting information

S1 Table Limits of detection (LOD) and quantification (LOQ) concentrations in the samples analyzed for microcystins (MC) and nodularin (NOD) by LC-MS/MS. (DOCX)

S2 Table Average (±SE) water-extracted concentrations of nutrients in pelican droppings. (DOCX)

S3 Table Average (±SE) concentrations of dissolved phosphate (PO4) and inorganic nitrogen (NO3+NH4) within the treatments after seven days of incubation. (n = 10).
(DOCX)
