# Supplementary material for: Effects of guanotrophication and warming on the abundance of green algae, cyanobacteria and microcystins in Lake Lesser Prespa, Greece
Source: PLoS One. 2020 Mar 11;15(3):e0229148. doi: 10.1371/journal.pone.0229148 (PMC7065754; doi:10.1371/journal.pone.0229148)
Supplement: S1 Table — (DOCX) [file pone.0229148.s001.docx]

| Compound | LOD^1^ (μg L^-1^) | LOQ^2^ (μg L^-1^) |
| --- | --- | --- |
| dm-7-MC-RR | 0.506 | 0.506 |
| MC-RR | 0.374 | 0.374 |
| NOD | < 0.034 | < 0.034 |
| MC-YR | < 0.048 | < 0.048 |
| dm-7-MC-LR | < 0.046 | < 0.046 |
| MC-LR | < 0.09 | < 0.09 |
| MC-LY | 0.79 | 0.79 |
| MC-LW | 0.538 | 0.538 |
| MC-LF | 0.226 | 0.226 |

^1^ Limit of detection, ^2^ Limit of Quantification
