# Supplementary material for: Effects of guanotrophication and warming on the abundance of green algae, cyanobacteria and microcystins in Lake Lesser Prespa, Greece
Source: PLoS One. 2020 Mar 11;15(3):e0229148. doi: 10.1371/journal.pone.0229148 (PMC7065754; doi:10.1371/journal.pone.0229148)
Supplement: S2 Table — (DOCX) [file pone.0229148.s002.docx]

|  |  | **Mean** | **SE** |
| --- | --- | --- | --- |
| **NO_3_** | mg N/g DW | 0.05 | 0.0 |
| **NH_4_** | mg N/g DW | 9.60 | 1.1 |
| **TIN** | mg N/g DW | 9.65 | 1.1 |
| **PO_4_** | mg P/g DW | 15.10 | 2.6 |
| **Al** | mg/g DW | 0.00 | 0.0 |
| **Ca** | mg/g DW | 7.22 | 1.1 |
| **Cl** | mg/g DW | 1.66 | 0.4 |
| **Fe** | mg/g DW | 0.00 | 0.0 |
| **K** | mg/g DW | 9.34 | 1.4 |
| **Mg** | mg/g DW | 2.49 | 0.2 |
| **Mn** | mg/g DW | 0.00 | 0.0 |
| **Na** | mg/g DW | 3.79 | 0.4 |
| **S** | mg/g DW | 7.11 | 0.8 |
| **Si** | mg/g DW | 0.05 | 0.0 |
