# Supplementary material for: Effects of guanotrophication and warming on the abundance of green algae, cyanobacteria and microcystins in Lake Lesser Prespa, Greece
Source: PLoS One. 2020 Mar 11;15(3):e0229148. doi: 10.1371/journal.pone.0229148 (PMC7065754; doi:10.1371/journal.pone.0229148)
Supplement: S3 Table — (n = 10). (DOCX) [file pone.0229148.s003.docx]

| **Site** | **Treatment** | **PO_4_ (µmol L^-1^)**  **20°C** | | **NO_3_+NH_4_ (µmol L^-1^) 20°C** | | **PO_4_ (µmol L^-1^)**  **30°C** | | **NO_3_+NH_4_ (µmol L^-1^) 30°C** | |
| --- | --- | --- | --- | --- | --- | --- | --- | --- | --- |
|  |  | Mean | *SE* | Mean | *SE* | Mean | *SE* | Mean | *SE* |
| **1** | **Control** | 0,2 | *0,10* | 68,4 | *13,6* | 0,3 | *0,03* | 47,1 | *28,0* |
|  | **+N** | 0,3 | *0,06* | 1073,4 | *11,6* | 0,4 | *0,00* | 1795,9 | *413,6* |
|  | **+P** | 25,6 | *1,96* | 35,6 | *8,2* | 22,4 | *2,01* | 93,8 | *22,9* |
|  | **+N+P** | 31,8 | *4,27* | 1026,2 | *46,5* | 24,7 | *0,77* | 2239,3 | *561,0* |
|  | **Dropping** | 15,4 | *1,35* | 231,3 | *32,5* | 8,5 | *0,15* | 212,8 | *14,2* |
| **2** | **Control** | 0,3 | *0,03* | 9,4 | *0,3* | 0,3 | *0,16* | 60,2 | *21,3* |
|  | **+N** | 0,4 | *0,00* | 902,7 | *21,9* | 0,2 | *0,00* | 1242,4 | *18,0* |
|  | **+P** | 23,8 | *0,48* | 18,2 | *7,0* | 41,2 | *3,36* | 32,0 | *7,2* |
|  | **+N+P** | 18,7 | *3,42* | 887,7 | *3,4* | 35,9 | *2,43* | 1122,6 | *109,7* |
|  | **Dropping** | 18,1 | *0,99* | 338,5 | *12,7* | 7,0 | *0,38* | 333,6 | *16,2* |
| **3** | **Control** | 0,1 | *0,03* | 45,9 | *13,1* | 0,2 | *0,06* | 61,3 | *13,0* |
|  | **+N** | 0,3 | *0,03* | 910,1 | *13,2* | 0,4 | *0,03* | 1495,5 | *23,4* |
|  | **+P** | 39,4 | *7,02* | 25,3 | *6,4* | 44,1 | *0,49* | 34,2 | *8,7* |
|  | **+N+P** | 48,8 | *0,96* | 985,4 | *29,2* | 27,0 | *1,30* | 1439,4 | *249,8* |
|  | **Dropping** | 25,6 | *1,82* | 315,0 | *23,7* | 7,0 | *0,31* | 298,8 | *11,4* |
